# The impact of parental psychopathology and sociodemographic factors in selective mutism - a nationwide population-based study

**DOI:** 10.1186/s12888-020-02637-6

**Published:** 2020-05-12

**Authors:** Miina Koskela, Roshan Chudal, Terhi Luntamo, Auli Suominen, Hans-Christoph Steinhausen, Andre Sourander

**Affiliations:** 1 Research Centre for Child Psychiatry, Institute of Clinical Medicine, Faculty of Medicine, University of Turku, Lemminkäisenkatu 3 / Teutori (3rd floor), 20014 Turku, Finland; 2 Turku University Central Hospital, Turku, Finland; 3 Department of Child and Adolescent Psychiatry, Psychiatric University Clinic, Zurich, Switzerland; 4 Clinical Psychology and Epidemiology, Department of Psychology, University of Basel, Basel, Switzerland; 5 Department of Child and Adolescent Psychiatry, University of Southern Denmark, Odense, Denmark; 6 Child and Adolescent Mental Health Centre, Capital Region Psychiatry, Copenhagen, Denmark; 7 Department of Child Psychiatry, Turku University Hospital, Turku, Finland; 8 INVEST Research Flagship, University of Turku, Turku, Finland

**Keywords:** Selective mutism, Parental age, Parental psychopathology, Prenatal factors, Epidemiology

## Abstract

**Background:**

Selective mutism (SM) is nowadays considered a relatively rare anxiety disorder characterized by children failing to speak in certain situations. Research on risk factors for SM are limited in comparison to other psychiatric disorders. The aim of this study was to examine several potential risk factors for SM in a large nationwide cohort, namely parental psychopathology, parental age, maternal SES, urbanicity, maternal marital status and parental immigration status.

**Methods:**

This nested case-control study comprised 860 cases with SM, identified from the Finnish Hospital Discharge Register and 3250 controls matched for sex and age from the Finnish Central Population Register. Conditional logistic regression was used to examine the association between the risk factors and SM.

**Results:**

If both parents had any psychiatric disorder, this almost tripled their odds of having a child with SM (OR 2.8, 95% CI 2.0–4.0). There were increased rates of all types of psychiatric disorders in the parents of the children with SM, with a wider range of diagnoses among the mothers than fathers. Fathers over 35 years (OR 1.4, 95% CI 1.1–1.8) were significantly more likely to have children with SM. Offspring of a single mother had a 2-fold (OR = 2.0, 95% CI 1.4–3.0) increased odds of SM than mothers who were married or in a relationship.

**Conclusions:**

Several parental psychiatric disorders were associated with offspring SM. This points towards a shared aetiology of psychiatric disorders. Findings on paternal age and single motherhood help to improve our understanding of risk factors for SM.

## Background

Selective mutism (SM) is nowadays considered an anxiety disorder that typically starts in childhood. According to the International Classification of Diseases, Tenth Revision (ICD-10), the disorder is called elective mutism, but because the term “selective mutism” has been established broadly, even in countries using the ICD-10 classification, the present study uses that term [1]. It is characterized by children failing to speak in certain situations where they are expected to speak, although they speak normally when they are in other situations. Problems can occur in a number of settings, such as when they are at school or when they are with unfamiliar people or in unfamiliar places.

The diagnostic criteria in the ICD-10 states that the term SM should be used if the symptoms appear consistently in specific social situations for a period of at least 1 month [1]. Furthermore, the child’s speech and language development should be within the normal range, the child should not fulfil the diagnostic criteria for pervasive developmental disorder (code F84) and SM should not be due to a lack of knowledge of the language being spoken. The Diagnostic and Statistical Manual of Mental Disorders, Fifth Edition (DSM-5) has some additional diagnostic criteria that requires that the disturbance interferes with educational achievements or social communication, is not explained by a communication disorder and does not occur exclusively during the course of schizophrenia or another psychotic disorder [2, 3]. SM often occurs in conjunction with anxiety disorders, especially social phobias, and the rates of comorbidity vary from 12 to 100%, depending on the diagnostic criteria that is used [2].

The etiology of SM includes various genetic, environmental and neurodevelopmental factors. Possible risk factors include problems in early development, shyness, anxiety, bilingualism and problems with speech and language development. Factors that might play a role in the onset of the disorder include moving and starting a new school or day-care center [2, 4]. At a behavioral level, SM has been associated with a controlling parenting style, due to child and parental anxiety, and the pattern of the symptoms are similar to anxiety disorders [5, 6]. Further knowledge of the aetiological factors is important to understand why the disorder occurs and how to prevent its onset.

Findings on the links between parental psychopathology and SM are not uniform. Two studies found associations between paternal psychopathology and SM, but not maternal psychopathology [7, 8]. A recent study found that the mothers of children with SM displayed more obsessive-compulsive features and fathers exhibited more phobic anxiety symptoms than the parents of children with generalized anxiety disorder [9]. There have been findings of higher rates of maternal schizoid and schizotypal features [10], increased levels of parental anxiety [8, 10] and social anxiety among the parents of children with SM [11].

There is only limited knowledge on the socio-demographic features associated with offspring SM. Parental age at the time of the child’s birth has been associated with a number of child and adolescent psychiatric disorders [12–15]. McGrath et al. [14] found an association with both low maternal age and advanced paternal age and behavioural and emotional disorders of childhood, which includes SM. To date, no previous study has examined the association between parental age and SM. The disorder has been reported to be more common in families with a migrant background [16, 17] and to be slightly more common in families with lower socioeconomic status (SES) [17]. However, SM has been observed in all social strata [18]. Marital conflict has been shown to be a risk factor for offspring SM, but studies on the topic are limited [16].. It is largely unknown whether or not urbanicity might increase the risk for SM, but it has been shown to be a risk factor for many mental disorders, such as schizophrenic, anxiety and substance use disorders [15, 19, 20].

There are a number of issues that should be considered when evaluating the results of earlier studies. Most studies on SM have been based on clinical samples with modest sample sizes of between 50 and 150 children. In addition, many studies have relied on teachers and parents to identify SM cases, increasing the risk of selection and reporting biases [7, 16]. Previous studies have also been limited by the lack of information on potential covariates.

The main aim of this study was to examine the associations between parental psychopathology and SM. In addition, we examined several potential risk factors for SM in a large nationwide cohort, namely parental age, maternal SES, urbanicity, maternal marital status and parental immigration status. To address the role of comorbidity, we also report comorbid diagnoses for the SM cases in the study sample. Based on previous findings showing higher rates of parental psychopathology in several different diagnostic categories [9–11, 21], the study hypothesis was that there would be higher rates of psychiatric morbidity among the parents of children with SM compared to the controls. In addition, we hypothesized that both advanced and young parental age would be associated with SM, as seen with behavioural and emotional disorders of childhood [14].

## Methods

### Registers

This nationwide register-based case-control study included information on all singleton children born alive in Finland between 1 January 1987 and 30 March 2009, who were diagnosed with SM between 1 January 1987 and 31 December 2016, as registered in the Finnish Hospital Discharge Register (FHDR). This study is part of a larger sample of anxiety disorders, the Finnish Prenatal study of Anxiety disorders (FIPS-Anxiety), which used data collected from three nationwide Finnish registers: The Finnish Hospital Discharge Register, the Finnish Central Population Register (FCPR) and The Finnish Medical Birth Register (FMBR).

The FHDR includes information on all visits to specialized health services. When a patient receives a diagnosis from one of these services, the relevant ICD-10 code is added to their medical records and that information is then recorded in the FHDR. The FHDR only contains inpatient records from 1969 to 1998 and both inpatient and outpatient diagnoses since that date. The FHDR was used to identify all SM cases that had been diagnosed by specialized mental health service, and to collect parental psychiatric diagnoses and comorbid diagnoses of the cases that were registered at least once during the observation period. The FHDR includes all diagnoses for patients who have received inpatient treatment in Finnish hospitals for physical and psychiatric conditions since 1969 and it also includes diagnoses from outpatient visits since 1998. Since 1996 the diagnoses have been coded using ICD-10 [1] and from 1987 to 1995 the Ninth Revision (ICD-9) codes were used [22].

The FCPR is a database that provides information about the person’s name and address, date of birth and death, family members and any immigration or emigration status. Information is available on all citizens and permanent residents in Finland, and it is maintained by the Population Register Centre and local register offices. This study used the FCPR to identify the fathers of the SM cases and controls and to collect data on the risk factors of paternal age, urbanicity and immigration.

The FMBR collects data during pregnancy and labour and has recorded information about all newborn infants in the country since 1987. In this study, the FMBR was used for information on maternal age and maternal marital status at the time of the child’s birth.

It was possible to link the information between these registers as each Finnish resident is issued with a personal identity code (PIC) at birth or when they move to the country. The study received approval from the data protection authority to use the health register data and link that data for the purposes of this study. Ethical approval for the study was provided by the Ethics Committee of the Hospital District of Southwest Finland.

Finnish hospital registers have been widely used for research and the overall quality of the registers has been found to vary from satisfactory to very good, depending on the diagnosis [23]. In particular, validation studies have shown that the diagnoses recorded in the FHDR have strongly met the diagnostic criteria for attention-deficit/hyperactivity disorder (ADHD) (88%), autism spectrum disorders (ASD) (95%) and Tourette’s syndrome (96%) [24–26].

### Diagnosing SM in Finnish healthcare

In Finland, health care services are publicly financed, and child health care is provided free of charge. Practically all children (99.5%) [27] in Finland attend general health check-ups, i.e. 15 check-ups before starting school at the age of seven, followed by annual check-ups. Registered nurses and doctors conduct these check-ups and a broad assessment of psychosocial well-being is carried out at the ages of four, seven, 11 and 14 years. If there are any concerns, the child is evaluated first by the primary health care personnel and then, if needed, referred to specialized health care services. One in eight children visit specialized health services for psychiatric or neurodevelopmental disorders by the age of 14 [28]. In specialized health services, all previous information on the child’s health is used to establish the diagnosis. The child is assessed by a doctor, and a psychologist if needed, and information from their day care or school and any parental assessments are taken into account. All diagnoses in Finland are made based on the ICD criteria and registered in the FHDR.

### Cases and controls

The study comprised a nested case-control sample that included all the cases collected for the cohort. The controls for each case were selected from the population at risk and matched to each case using selected factors. The cases were identified from the FHDR using the ICD-10 code F94.0 and the ICD-9 code 3132C and this resulted in a total sample of 1114 children. The study included cases that were diagnosed at least once after the age of three and before the age of 15. The exclusion criteria were coexisting ASD (ICD-10 F84.0–.9, ICD-9229), moderate or severe intellectual disability (ICD-10 F72–73, ICD-9318) and psychotic disorders diagnosed before or concurrently with SM (ICD-10 F20–25, F28–29, ICD-9295, 297, 2989X and 3012C). There were 860 cases in the final analyses.

All SM cases were matched with four controls by their birth date (±30 days) and sex. As the original FIPS-Anxiety project had predefined inclusion and exclusion criteria for cases and matched controls, the controls in this SM study sample could not have any anxiety disorders. This meant that 143 controls with diagnoses of anxiety and childhood emotional disorders (ICD-10 codes F40–42, F43.0, F43.1, F43.22, F43.23 and F93–94) were excluded. Additional exclusion criteria for the controls were ASD and moderate or severe intellectual disability. A total of 3250 controls were finally included in the analyses. Table S1 (Additional file 1) shows a flow chart for the included and excluded cases.

### Validation of SM diagnosis

We searched the patient record system of the Hospital District of Southwest Finland, which provides secondary and tertiary level treatment to around 200,000 residents [29], following approval from the hospital’s ethics committee and the Turku Clinical Research Centre. This search identified 53 children diagnosed with SM with the ICD-10 code F94.0. The birth years and exclusion criteria of the cases were the same as in the epidemiological study.

Two reviewers (PJ, MK) performed chart assessments for all 53 cases, namely a medical doctor who specialized in forensic psychiatry and a fifth-year medical student. Two experienced specialists in child and adolescent psychiatry (AS2, TL) supervised the process. The medical records we used were evaluated using ICD-10 criteria [1]. To determine the final case status, the detailed recordings from each reviewer were compared and reviewed by a specialist in child psychiatry (TL).

### Risk factors

The risk factors that were examined were maternal and paternal psychopathology, maternal and paternal age, maternal SES, maternal marital status, parental immigration status and urbanicity. Parental psychiatric diagnoses were based on ICD-9 and ICD-10 codes. These were divided into 14 categories based on ICD-10 definitions, as follows: SM, ADHD, ASD, conduct disorders, learning and coordination disorders, intellectual disabilities, schizophrenia and schizoaffective disorders, other non-affective psychoses, bipolar disorders, unipolar mood disorders, anxiety disorders, personality disorders, alcohol and drug addiction/abuse and other psychiatric disorders. The ICD-10 and ICD-9 codes that were used are described in the Additional file 1 (Table S2).

In line with earlier studies [30], if a parent was diagnosed with schizophrenia or a schizoaffective disorder, he or she would not be assigned to any other additional adolescent or adult-onset categories, because these are distinctively severe and chronic disorders. Other parents could have belonged to several diagnostic categories. In addition, a category called any parental psychopathology was created, meaning that the parent had at least one psychiatric disorder. This was further divided into three subcategories: mother only, father only and both parents having at least one psychiatric diagnosis. Subsequently, separate diagnostic categories were used to examine any associations between parental psychopathology and SM.

Maternal and paternal ages were categorized into six groups, namely: less than 20, 20–24, 25–29, 30–34, 35–39 and 40 or more years. Information on maternal age was available for all the cases and controls, but the data on 19 (2.2%) of the fathers of children with SM and 31 (1.0%) of the control fathers were not available. Maternal SES was divided into the five categories of maternal occupation used by Gissler et al. (2003), namely: upper white collar, lower white collar, blue collar, other and missing. These categories were based on the recommendations by Statistics Finland to divide different occupations or education levels into previously used social classes. The other class included occupations that could not be classified into white or blue collar, like students, farmers, entrepreneurs and housewives [31, 32]. Maternal SES, derived from occupational class, has been shown to be a stronger indicator of health inequality in Finland than paternal SES [33]. Furthermore, paternal SES is not recorded in the registers in Finland and this information is therefore unavailable. Marital status was classified as married/in a relationship or single. An immigrant was defined as a person who was born abroad and was not a native Finnish speaker. Those who were born in Finland and/or whose native language was one of Finland’s official languages, i.e. Finnish, Swedish, or Sami, were defined as Finnish. Immigration status was divided into both parents being immigrants, only the mother being an immigrant, only the father being an immigrant and both parents being Finnish. The location of the family home was described using the Statistics Finland categories, which are based on the density of the population in that area: urban, semi-urban or rural [32, 34].

### Psychiatric comorbidity

The FHDR was used to obtain all comorbid psychiatric diagnoses among SM cases. Comorbidity was defined as being given an additional psychiatric or neurodevelopmental disorder diagnosis at least once during the observation period. The detailed classification of the diagnostic codes used for assessing comorbidity is presented in the Additional file 1 (Table S3).

### Statistical analyses

The primary outcome of the analysis was a diagnosis of SM. The risk factors that were examined included: any maternal and paternal psychopathology, maternal and paternal age, maternal SES, maternal marital status, parental immigration status and urbanicity. To assess covariates, Pearson’s chi-square test was used to examine the association between each primary risk factor and the other listed risk factors as potential covariates. Conditional logistic regression was used to examine the associations between the other risk factors and SM. All risk factors were tested for associations with each other as primary risk factors.

Conditional logistic regression was used to examine the unadjusted association between primary risk factors and SM and then adjustments were made for all significant covariates. The final multivariate model contained all statistically significant factors (*p* < 0.10). The relationships between the exposures and SM status were reported as odds ratios (OR) with 95% confidence intervals (CI), together with *P*-values. Two-sided P-values of less than 0.05 were deemed statistically significant.

Cases with SM were stratified into two groups for an additional analysis: all SM cases irrespective of comorbid psychiatric diagnoses and SM without any comorbid psychiatric disorders (ICD-10 F10-F99, ICD-9291–319, excluding 316). Conditional logistic regression was used to examine any associations between examined risk factors and the stratified groups.

Several sensitivity analyses were conducted to test for the possible effects of including SM diagnoses before 1998, which only covered inpatient care. From 1998 the register also included outpatient diagnoses. First, we excluded all the SM cases that were only diagnosed before 1998 and conducted conditional logistic regression analyses, without their matched strata, to see whether all the same risk factors remained significant. Then parental psychiatric diagnoses before 1998 were excluded from the conditional regression analyses to see whether the diagnostic groups that were significant in the original data retained that significance. Finally, a similar comparison was conducted after SM cases diagnosed before 1998, with their matched strata, and parental psychiatric diagnoses before 1998 were excluded from the conditional logistic regression analyses. The statistical analyses were performed with SAS software, version 9.4 (SAS Institute Inc., Cary, NC, USA).

## Results

The study comprised 860 children with SM (59.1% girls) and 3250 controls (58.9% girls). The prevalence of SM by the end of the follow-up period was 6.2 per 10,000. The mean ages, standard deviations and ranges at the first diagnosis were 8.1 ± 3.1 years for girls (3–15 years) and 7.9 ± 3.0 for boys (3–15 years). The means, standard deviations and ranges of the maternal ages at their child’s birth were 29.2 ± 5.8 (16–45) years for the SM sample and 29.1 ± 5.3 (15–48) years for the controls. The respective ages for the fathers were 32.4 ± 6.5 (19–57) years and 31.6 ± 6.1 (16–61) years. As shown in Table 1, more than two-thirds (69.1%) of the children with SM had been given an additional psychiatric or neurodevelopmental disorder diagnosis at least once during the observation period. The most common comorbidities were learning and coordination disorders (33.1%), childhood emotional disorders (23.0%) and affective disorders (19.0%). The study found that 5.4% of the cases had childhood onset social phobias and that a further 5.5% had social phobias diagnosed in adolescence or adulthood.

**Table 1:** Comorbidity among children with selective mutism during the follow-up period

	Cases N (%)	Controls N (%)
**1.Any psychiatric or neurodevelopmental disorder**	594 (69.1)	3766 (11.3)
**a. Schizophrenia spectrum disorders**	27 (3.1)	11 (0.3)
**b. Affective disorders**	163 (19.0)	94 (2.9)
***Bipolar disorders: F30, F31)***	5 (0.6)	9 (0.3)
***Unipolar disorders: F32, F33, F34, F38, F39***	162 (18.8)	89 (2.7)
**c. Anxiety disorders**	143 (16.6)	*excluded*
***F40 Phobic anxiety disorders***	73 (8.5)	*excluded*
***F40.1 Social anxiety disorder***	47 (5.5)	*excluded*
***F41 Other anxiety disorders (excluding F41.2)***	74 (8.6)	*excluded*
***F42 Obsessive– compulsive disorder***	26 (3.0)	*excluded*
**d. Other neurotic and personality disorders**	139 (16.2)	75 (2.3)
**e. Substance abuse disorder**	23 (2.7)	39 (1.2)
**f. Attention deficit hyperactivity disorder**	51 (5.9)	48 (1.5)
**g. Intellectual disability**^a^	42 (4.9)	14 (0.4)
**h. Childhood emotional disorders**	198 (23.0)	*excluded*
***F93.0 Separation anxiety***	23 (2.7)	*excluded*
***F93.1 Childhood onset phobic and anxiety disorders***	22 (2.6)	*excluded*
***F93.2 Childhood onset social anxiety disorder***	46 (5.4)	*excluded*
***F93.3–9 Other childhood onset emotional and anxiety disorders***	140 (16.3)	*excluded*
**i. Conduct and oppositional disorders**	104 (12.1)	28 (0.9)
**j. Tic disorders**	12 (1.4)	9 (0.3)
**k. Learning and coordination disorders**	285 (33.1)	161 (5.0)
***F80 Specific developmental disorders of speech and language***	191 (22.1)	84 (2.6)
***F81 Specific developmental disorder of scholastic skills***	70 (8.1)	57 (1.8)
***F82 Specific developmental disorder of motor function***	44 (5.1)	28 (0.9)
***F83 Mixed specific developmental disorder***	78 (9.1)	26 (0.8)

^a^Moderate and severe intellectual disability were excluded

Covariate testing revealed that there were significant correlations between both maternal and paternal psychopathology and SM, with regard to parental age, maternal SES and the psychopathology of the other parent. The detailed distribution of the covariate data and test findings are presented in the Additional file 1 (Tables S4-S7). The covariates were based on this analysis. In the adjusted analyses of parental psychopathology, maternal psychopathology was adjusted with paternal psychopathology, maternal SES and parental age. Paternal psychopathology was adjusted with maternal psychopathology, maternal SES and parental age.

Table 2 shows the associations between parental psychopathology and offspring SM. In the adjusted analyses, having a mother (*N* = 163, OR 2.0, 95% CI 1.6–2.5) or a father (*N* = 108, OR 1.7, 95% CI 1.4–2.2) with any psychiatric diagnosis significantly increased the odds of SM in the child. The association was strongest when both parents had a psychiatric diagnosis (*N* = 69, OR 2.8, 95% CI 2.0–4.0). The odds for both parents having a psychiatric diagnosis was significantly stronger than only a father (OR 1.7, 95% CI 1.1–2.5, *P* = 0.0077) or only a mother having a psychiatric diagnosis (OR 1.5, 95% CI 1.01–2.1, *P* = 0.042).

**Table 2:** Association between any parental psychopathology and offspring SM

Parental psychopathology^**a**^	Cases (***N*** = 860) N (%)	Controls (***N*** = 3250) N (%)	Unadjusted OR (95% CI)	Adjusted OR (95% CI)
None	501 (59.6)	2425 (75.3)	*Reference*	*Reference*
Both	69 (8.2)	108 (3.4)	3.0 (2.2–4.2)***	2.8 (2.0–4.0)***
Mother only	163 (19.4)	392 (12.2)	2.0 (1.6–2.5)***	2.0 (1.6–2.5)***
Father only^**a**^	108 (12.8)	294 (9.1)	1.8 (1.4–2.3)***	1.7 (1.4–2.2)***

Odds ratios (OR) were adjusted for socioeconomic status, maternal age and paternal age.

^**a**^Missing paternal data for 19 cases (2.2%) and 31 controls (1.0%)

*** *P*-value< 0.0001

The associations between specific parental diagnoses and child SM are shown in Table 3. After adjustment, there were significantly increased odds with regard to all maternal psychiatric diagnoses, except for SM, ASD, ADHD, conduct and oppositional defiant disorders and learning and coordination disorders. In particular, schizophrenia and schizoaffective disorders (*N* = 18, OR 4.4, 95% CI 2.0–10.1), other maternal psychoses (*N* = 35, OR 4.2, 95% CI 2.4–7.2), and personality disorders (*N* = 48, OR 3.3, 95% CI 2.1–5.1) were strongly associated with offspring SM. For paternal disorders, significantly increased odds were limited to other psychoses (*N* = 17, OR 2.1, 95% CI 1.1–4.1), unipolar mood disorders (*N* = 68, OR 1.5, 95% CI 1.1–2.0), personality disorders (*N* = 43, OR 1.9, 95% CI 1.3–2.8), alcohol and drug addiction/abuse (*N* = 79, OR 1.9, 95% CI 1.4–2.5) and learning and coordination disorders (*N* = 5, OR 5.6, 95% CI 1.3–25.0).

**Table 3:** Association between parental psychiatric diagnoses and offspring SM

	Mothers			
	Cases (***N*** = 860) N (%)	Controls (***N*** = 3250) N (%)	OR (95% CI)	Adjusted OR^**a**^ (95% CI)
**Schizophrenia and** **schizoaffective disorders**	18 (2.1)	14 (0.4)	5.0 (2.4–10.2)***	4.4 (2.0–10.1)**
**Selective mutism**	0 (0.0)	0 (0.0)	N/A	N/A
**Other psychoses**	35 (4.1)	28 (0.9)	4.7 (2.9–7.8)***	4.2 (2.4–7.2)***
**Bipolar disorders**	17 (2.0)	24 (0.7)	2.8 (1.5–5.2)*	2.2 (1.1–4.2)*
**Unipolar mood disorders**	158 (18.4)	276 (8.5)	2.4 (2.0–3.0)***	2.2 (1.7–2.7)***
**Anxiety disorders (including childhood anxiety disorders)**	66 (7.7)	112 (3.5)	2.4 (1.7–3.3)***	2.1 (1.5–3.1)***
**Personality disorders**	48 (5.6)	51 (1.6)	3.7 (2.4–5.6)***	3.3 (2.1–5.1)***
**Alcohol and drug addiction/abuse**	43 (5.0)	84 (2.6)	2.0 (1.4–2.9)**	1.7 (1.2–2.6)*
**Attention-deficit/** **hyperactivity disorders**	3 (0.4)	11 (0.3)	1.0 (0.3–3.7)	1.3 (0.4–4.9)
**Autism spectrum disorders**	0 (0.0)	1 (0.03)	N/A	N/A
**Conduct/oppositional defiant disorders**	1 (0.1)	3 (0.1)	N/A	N/A
**Learning and** **coordination disorders**	5 (0.6)	3 (0.1)	6.4 (1.5–27.0)*	4.0 (0.9–17.7)
**Intellectual disability**	5 (0.6)	2 (0.1)	9.6 (1.9–49.7)*	7.9 (1.4–43.2)*
**Other psychiatric disorders**	97 (11.3)	257 (7.9)	1.5 (1.2–1.9)*	1.4 (1.06–1.8)*
	**Fathers**			
	**Cases (*N*** **= 841)** **N (%)**	**Controls (*N*** **= 3219)** **N (%)**	**OR** **(95% CI)**	**Adjusted OR**^**b**^ **(95% CI)**
**Schizophrenia and** **schizoaffective disorders**	8 (1.0)	11 (0.3)	2.7 (1.1–6.7)*	2.1 (0.8–5.4)
**Selective mutism**	0 (0.0)	0 (0.0)	N/A	N/A
**Other psychoses**	17 (2.0)	27 (0.8)	2.6 (1.4–4.9)*	2.1 (1.1–4.1)*
**Bipolar disorder**	14 (1.7)	35 (1.1)	1.6 (0.8–3.0)	1.6 (0.8–3.1)
**Unipolar mood disorders**	68 (8.1)	153 (4.8)	1.8 (1.3–2.4)**	1.5 (1.1–2.0)*
**Anxiety disorders (including childhood anxiety disorders)**	37 (4.4)	81 (2.5)	1.8 (1.2–2.7)*	1.5 (0.98–2.3)
**Personality disorders**	43 (5.1)	77 (2.4)	2.2 (1.5–3.2)***	1.9 (1.3–2.8)*
**Alcohol and drug addiction/abuse**	79 (9.4)	143 (4.4)	2.2 (1.7–3.0)***	1.9 (1.4–2.5)***
**Attention deficit/** **hyperactivity disorders**	3 (0.4)	4 (0.1)	3.0 (0.7–13.4)	2.2 (0.5–10.4)
**Autism spectrum disorders**	3 (0.4)	1 (0.03)	N/A	N/A
**Conduct/oppositional defiant disorders**	3 (0.4)	3 (0.1)	3.8 (0.8–18.9)	2.9 (0.6–14.8)
**Learning and** **coordination disorders**	5 (0.6)	3 (0.1)	6.4 (1.5–27.0)*	5.6 (1.3–25.0)*
**Intellectual disability**	2 (0.2)	3 (0.1)	2.7 (0.4–16.0)	1.4 (0.2–9.9)
**Other psychiatric disorders**	57 (6.8)	165 (5.1)	1.4 (0.99–1.86)	1.2 (0.9–1.7)

^**a**^Adjusted for maternal socioeconomic status (SES), maternal and paternal age and paternal psychopathology

^**b**^Adjusted for maternal SES, maternal and paternal age and maternal psychopathology

Cases without any specific diagnoses in each group were used as reference values.

*P ≤ 0.05 **P ≤ 0.001 ***P < 0.0001

Finally, the associations between parental age, SES, marital status, immigration and urbanicity and offspring SM are presented in Table 4. In the adjusted analyses, children born to fathers who were over the age of 35 years had 1.4-fold (*N* = 178, 95% CI 1.1–1.8) odds of SM, and it was 1.8-fold (*N* = 123, 95% CI 1.4–2.4) when the father was over the age of 40. Increased odds were also seen among children born to mothers aged 20–24 years (*N* = 174, OR 1.4, 95% CI 1.1–1.7), but the significance was lost after adjusting for covariates (OR 1.2, 95% CI 0.9–1.6). When the highest SES class was used as the reference, all other maternal SES classes displayed increased odds of SM and the highest odds were seen with the lowest defined category, namely blue collar workers (*N* = 188, OR 2.8, 95% CI 2.0–4.0). In the adjusted analyses, single mothers had 2.0-fold odds (*N* = 55, 95% CI 1.4–3.0) of having a child with SM than mothers who were married or in a relationship. There were no significant associations between parental immigration status or urbanicity and offspring SM.

**Table 4:** Association between sociodemographic factors and offspring SM

	Cases (***N*** = 860) N (%)	Controls (***N*** = 3250) N (%)	Unadjusted OR (95% CI)	Adjusted OR (95% CI)
**Paternal age**^**a**^	***N*** **= 841**	***N*** **= 3219**		
< 20	7 (0.8)	21 (0.7)	1.3 (0.6–3.2)	0.95 (0.4–2.3)
20–24	85 (10.2)	335 (10.4)	1.0 (0.8–1.4)	0.9 (0.6–1.2)
25–29	210 (25.0)	874 (27.2)	*reference*	*reference*
30–34	238 (28.3)	1103 (34.3)	0.9 (0.7–1.1)	1.01 (0.8–1.2)
35–39	178 (21.2)	578 (18.0)	1.3 (1.03–1.6)*	1.4 (1.1–1.8)*
≥40	123 (14.6)	308 (9.6)	1.7 (1.3–2.2)**	1.8 (1.4–2.4)**
**Maternal age**^**b**^	***N*** **= 860**	***N*** **= 3250**		
< 20	27 (3.1)	89 (2.7)	1.3 (0.8–2.1)	1.1 (0.6–2.0)
20–24	174 (20.2)	543 (16.7)	1.4 (1.1–1.7)*	1.2 (0.9–1.6)
25–29	261 (30.4)	1114 (34.3)	*reference*	*reference*
30–34	222 (25.8)	969 (29.8)	0.98 (0.8–1.2)	0.9 (0.7–1.1)
35–39	137 (15.9)	442 (13.6)	1.3 (1.1–1.7)*	0.9 (0.7–1.3)
≥40	39 (4.5)	93 (2.9)	1.8 (1.2–2.7)*	1.1 (0.6–1.8)
**Maternal socioeconomic status**^**c**^	***N*** **= 860**	***N*** **= 3250**		
Upper white collar	67 (7.8)	495 (15.2)	*reference*	*reference*
Lower white collar	296 (34.4)	1183 (36.4)	2.0 (1.4–2.7) ***	2.0 (1.5–2.7)***
Blue collar	188 (21.9)	518 (15.9)	2.8 (2.0–3.9)***	2.8 (2.0–4.0)***
Other	166 (19.3)	508 (15.6)	2.3 (1.6–3.2)***	2.4 (1.7–3.4)***
Missing	143 (16.6)	546 (16.8)	2.3 (1.5–3.5)**	2.4 (1.5–3.6)***
**Marital status**^**d**^	***N*** **= 797**	***N*** **= 3005**		
Married/in a relationship	742 (93.1)	2911 (96.9)	*reference*	*reference*
Single	55 (6.9)	94 (3.1)	2.3 (1.6–3.3)***	2.0 (1.4–3.0)**
**Immigration status**^**e**^	***N*** **= 860**	***N*** **= 3250**		
Both parents Finnish	815 (94.77)	3070 (94.5)	*reference*	*reference*
Mother immigrant	19 (2.2)	50 (1.5)	1.5 (0.8–2.5)	1.4 (0.8–2.6)
Father immigrant	12 (1.4)	66 (2.0)	0.7 (0.4–1.3)	0.7 (0.4–1.4)
Both parents immigrants	14 (1.6)	64 (2.0)	0.8 (0.5–1.5)	0.7 (0.4–1.4)
**Urbanicity**^**f**^	***N*** **= 857**	***N*** **= 3222**		
Urban	523 (61.0)	1970 (61.1)	0.9 (0.8–1.1)	0.98 (0.8–1.2)
Semi-urban	132 (15.4)	550 (17.1)	0.8 (0.7–1.1)	0.8 (0.6–1.1)
Rural	202 (23.6)	702 (21.8)	*reference*	*reference*

^a^Odds ratio (OR) adjusted for maternal socioeconomic status (SES), maternal and paternal psychopathology)

^b^OR psychopathology adjusted for paternal age, maternal SES. marital status, maternal and paternal psychopathology

^c^OR adjusted for paternal age, marital status, maternal and paternal psychopathology

^d^OR adjusted for paternal age, maternal SES, and maternal and paternal psychopathology

^e^OR adjusted for paternal age, maternal SES, marital status, maternal and paternal psychopathology

^f^OR adjusted for paternal age, maternal SES, maternal and paternal psychopathology

Paternal age missing in 19 cases (2.2%) and 31 controls (1.0%).

Urbanicity missing in three cases (0.4%) and 28 controls (0.9%).

Marital status missing in 63 cases (7.3%) and 245 controls (7.5%).

* *P*-value≤0.05 **P-value≤0.001 *** *P*-value< 0.0001

Additional analyses examined potential interactions between risk factors and case-control status when comorbidities were present. The latter included all psychiatric disorders. There were no significant interactions between risk factors and case-control status for these comorbidities, as all the *P*-values were greater than 0.05. The results of the sensitivity analyses can be seen in Additional file 1 (Table S8).

Several sensitivity analyses were conducted on the findings for parents and children diagnosed since 1998. The results of these analyses can be seen in the Additional file 1 (Tables S9-S11). Tests carried out after we excluded the subgroup of 18 SM cases that were only clinically diagnosed before 1998 (*N* = 18), showed that the same risk factors remained significant as when they were included. After we excluded parental psychiatric diagnoses before 1998, all the parental psychopathology findings remained significant, except for paternal anxiety. After we excluded both child and parental diagnoses before 1998, the differences in paternal anxiety disorders were not statistically significant. In addition, the number of mothers with intellectual disabilities was too small for further analyses.

In the validation subsample, we were able to validate 52 of the 53 cases identified from the medical records, as one case did not have complete information. The full ICD-10 diagnostic criteria for SM were fulfilled in 45 of the 52 cases (87%). Two cases (4%) did not fulfil the diagnostic criteria, speech and language development were not within the normal range in four cases (8%) and one child (2%) fulfilled the diagnostic criteria for Asperger’s syndrome, which is an exclusion criterion for SM in ICD-10. However, all these five children (10%) displayed the core symptom of SM, namely clear muteness in situations where they were expected to speak, despite their ability to speak in other situations.

## Discussion

To our knowledge, this was the first register-based study to investigate the association between a wide range of parental psychopathologies and offspring SM using a case-control design. The main findings of our study were the different impact of maternal and paternal psychopathology on offspring SM and the association with high paternal age. In addition, being a single mother and having a lower SES raised the odds for offspring SM. The results of the sensitivity analyses performed by comparing subgroups with, and without, any comorbidities implied that the risk factors were associated with SM and not just with comorbid diagnoses. In addition, the results provide further insights into the impact of various risk factors on SM and point to a clustering of psychiatric disorders in the parents and to similar risk factors to those seen in other childhood psychiatric disorders [12, 14, 35]. Our findings on parental psychopathology are similar to findings related to several other disorders [30, 35]. In contrast, our findings on parental age partly differed from previous findings on behavioural and emotional disorders of childhood, as they showed no association between SM and low maternal age [14]. There was no association between urbanicity and SM in our study, which differed from the association with anxiety disorders reported by another study [19].

As hypothesized, both the mothers and fathers of the cases had significantly more psychiatric diagnoses than the parents of the controls. There were significantly greater odds of offspring SM if both parents had a psychiatric diagnosis, than if just the father or just the mother had a psychiatric disorder. The parents of the SM cases demonstrated higher rates of all types of disorders than the parents of the controls, but the range of diagnoses was wider among the mothers than the fathers. Therefore, this study points to a stronger impact of maternal than paternal psychopathology on offspring SM.

However, it should be noted that the majority of the parents (59.6%) of the cases did not have any psychiatric diagnoses. It is important to remember that, even though the mothers’ psychotic disorders were significantly associated with offspring SM, only 18 (2.1%) mothers had schizophrenia or a schizoaffective disorder and 35 (4.1%) were diagnosed with some other psychotic disorder.

Our results are in line with the findings of a number of previous studies. A study of 45 SM cases indicated that most of their parents had psychiatric symptoms [21]. A study of 50 cases by Kristensen et al. [10] found that there were symptom traits in both parents, showing significant associations for schizotypal features in mothers and anxiety traits in fathers. Furthermore, parental psychopathology has been shown to predict childhood psychopathology in various diagnoses [12, 15, 20]. However, these results are in contrast to other studies, which only found associations between paternal psychopathology and offspring SM [7, 8]. The differences between the studies may be due to different study designs and sample sizes, which varied from 26 to 70. Furthermore, some studies only found associations for specific psychopathological features, such as higher rates of maternal schizoid and schizotypal features [10], increased levels of parental anxiety [8, 10] and social anxiety among the parents of children with SM [11]. It has been suggested that the structure of diverse psychopathologies may best be explained by a general psychopathology dimension called the p-factor [36] and this factor could have also determined the risk factors in the present study. In addition, several studies have documented familial aggregation of various psychiatric disorders. This implies that having a certain psychiatric disorder could pose a general risk factor for family members to develop another psychiatric disorder [19, 37, 38]. However, the sensitivity analyses in the present study showed that the risk factors were not determined by comorbid disorders.

In addition to shared genetic factors, the relatively stronger association between maternal psychopathology and offspring SM could have been due to non-genetic perinatal factors, such as prenatal maternal medication use, substance use or nutritional status, obstetric complications and maternal stress during pregnancy. Evidence on the effects of obstetric complications on SM have been reported by one clinical study [17] and the potential impact of prenatal and perinatal risk factors on SM certainly needs further research. Maternal psychopathology could also affect the child’s psychosocial environment and the mother’s caregiving behavior. Parents of children with SM have been reported to be more anxious and controlling in social situations than parents of controls without SM [5], but self-reported parenting attitudes or strategies did not differ between the two groups [8, 39].

This was the first study to examine the association between parental age and SM. The novel finding was that increased paternal age at birth was associated with offspring SM, with the highest odds of 1.8-fold being reported for fathers over the age of 40. In contrast to the study hypothesis, young parental age and advanced maternal age were not associated with offspring SM. The findings of this study on SM were in line with studies on other mental disorders, which showed that advanced paternal age was associated with increased psychiatric morbidity in the offspring. This trend has already been shown for autism and ADHD [13, 40, 41], behavioural and emotional disorders [14] and schizophrenia [15]. There are several theories that could explain the association between increased paternal age and psychiatric disorders. First, the number of de novo mutations rise as the father’s age increases [42]. Second, epigenetic dysregulation due to increased age could lead to the development of psychiatric disorders [43]. Lastly, some older first-time fathers may also have lacked the social skills and personality traits needed to find partners or become parents at an earlier age [44]. Similar effects could be present with SM, as the parents of children with SM have been reported to be quieter and shyer than parents whose children do not have SM [10, 45]. However, it should be noted that advanced paternal age is not a universal risk factor for mental health disorders in offspring. For example, a Danish study of three generations did not find any effects of paternal age on bipolar disorders [46], anxiety disorders [19], obsessive compulsive disorder [12], phobic disorders [37] and substance use disorders [20]. Since this was the first study on parental age and SM, there is need to replicate the results in future studies with different samples.

Other sociodemographic factors examined in the present study as possible risk factors for offspring SM were maternal SES, maternal marital status, parental immigration status and urbanicity. No significant association was seen between parental immigration status and offspring SM. This finding was in contrast to previous studies, where SM was found to be more common in immigrant populations [16]. In 2017, the immigration rate in Finland was 4.5%, which was similar to the immigration rates of the cases (5.2%) and controls (5.5%) in our study [32]. Our findings on maternal SES were in line with previous literature. SM was seen in all social strata, and the lowest SES class, blue collar workers, had increased odds of having a child with SM when the highest SES class was used as a reference. [17, 18] Single mothers had an increased risk of having a child with SM. This was similar to a previous study that found that marital conflict was a risk factor for SM [16]. No associations were seen between urbanicity and SM. These findings indicate that psychosocial disadvantage might be a risk for offspring SM.

The additional validation study showed that the overall validity of the diagnosis was very satisfactory, as 87% of a local subsample of 52 cases were correctly diagnosed with SM. The core symptom of failure to speak in certain situations was even present in the rest of the cases that did not qualify for a definite diagnosis of SM. It may well be expected that the validity of the diagnosis was rather similar in the total sample of SM cases.

Finally, although register-based studies yield large sample sizes, the representativeness of the sample must be discussed. The mean age of SM at first diagnosis that is usually reported in the literature is 6.5–9 years [47], and it was 8.1 years in the present study. However, the age range at diagnosis, 3–15 years, was wider than in most clinical studies with much smaller sample sizes. The present study may well have included both more severe cases with early manifestations and chronic cases, which had been referred and diagnosed rather late. The mean age at first diagnosis was soon after starting school (which is age of seven in Finland), which is when teachers and parents are most likely to notice less severe manifestations of SM.

Before 1998 the register data only included inpatient diagnoses, which means that less severe SM cases were probably missing from the data during that time period. Sensitivity analyses showed that the lack of outpatient data before 1998 in the register did not result in bias. Furthermore, the rate of comorbid disorders of SM in the present study differed from previous findings, especially the low rate of social anxiety disorder. Wide variations have been reported in the rates of SM comorbid with social anxiety disorder, ranging from 12 to 100% [2], and there have been a lack of guidelines for differentiating between the two disorders in the ICD-10. With these factors in mind, the comorbidity rate of 5.4% with childhood onset social anxiety and 5.5% with adolescence or adulthood onset anxiety disorder in the present study may simply reflect varying diagnostic standards in a large national group of clinicians. The true overlap and differentiation of the two disorders needs to be established in future by carefully designed and controlled studies. Sensitivity analyses stratifying the sample by comorbid diagnoses showed no significant differences overall in both groups, indicating that all associations with risk factors were independent of comorbidities in SM.

### Strengths and limitations

The present study was based on the largest nationwide sample of children with SM to date. Register-based data is unlikely to be biased by recording differences, loss of follow up or selection of cases. We were able to investigate a wide spectrum of risk factors, using register-based data in a case-control setting with a ratio of 1:4. The analyses were statistically adjusted for several covariates. To our knowledge, the association between parental age and SM has not been investigated before.

Despite several strengths, such as the large sample size and robust methods and design, the study does have some limitations that need to be considered when interpreting the findings. Children with SM were identified from the Discharge Register, which is primarily used for clinical diagnoses and treatment in Finland. The validity of the diagnoses in the Finnish registry has been shown to be good for several disorders, including ASD, Tourette’s Syndrome and ADHD [24–26]. The validation of the SM diagnosis was also satisfactory in the present study. Furthermore, less severe cases of SM were not necessarily referred to specialized services, in common with all mental health disorders, and they were less likely to be noticed by parents or teachers. It is likely that data were missing for some parental diagnoses of both cases and controls not requiring inpatient treatment before 1998, such as anxiety disorders and SM. Sensitivity analyses were conducted to control for the effect of only including inpatient diagnoses before 1998. The results showed that all the same risk factors, and all but two of the parental diagnosis categories, remained significant once diagnoses before 1998 were excluded. However, it is important to bear in mind that only 18 cases were diagnosed before 1998 and this small number could make the results of the sensitivity analyses uncertain. The present study is a subsample of the FIPS-anxiety study. It was based on predesigned inclusion and exclusion criteria and this meant that we had to exclude all controls with anxiety disorders. Even though sensitivity analyses for comorbidities did not result in significant findings, the study might have profited from a closer matching of cases and controls with regard to comorbidities. However, the pre-designed frame of the FIPS-project did not allow for this option. Therefore, it was not possible to compare comorbidities between cases and controls in the analyses. Finally, the overall rates of immigration in Finland is low and immigrants only accounted for 5.2% of the cases in this study. This means that these results might not be generalizable to populations with greater ethnic and cultural diversity.

## Conclusions

There has been limited research on SM, compared with most of the other childhood onset disorders, and no previous nationwide population-based research. This is of concern, due to the chronicity, disease burden, and psychosocial impairment of SM. Several findings from this study may contribute to our understanding of parental risk factors and SM. This study showed that SM was associated with parental psychopathology and a cluster of several parental psychiatric disorders. This clustering points towards some shared origins with other psychiatric disorders. In particular, the association with maternal psychopathology may imply that additional prenatal and postnatal environmental risk factors also had an impact. This assumption might have important implications for future research. Furthermore, this was the first study to explore the association between parental age and SM. The present findings are important as they may contribute to earlier detection and treatment of SM. When clinicians are assessing children with SM, particularly their family history, they need to be aware of various kinds of preceding and/or concurrent parental psychopathology that might provide risk factors that are relevant to the aetiology of the child’s disorder. Furthermore, these findings need to be considered when planning the child’s treatment.

## Supplementary information

**Additional file 1: Supplementary Table S1.** Inclusion and exclusion criteria of Selective Mutism. **Supplementary Table S2.** Diagnostic categories and codes for parental psychiatric disorders. **Supplementary Table S3.** Diagnostic categories and codes for comorbid psychiatric disorders in children. **Supplementary Table S4**. Covariate testing for maternal psychopathology. **Supplementary Table S5.** Covariate testing for parental psychopathology. **Supplementary Table S6.** Covariate testing for paternal age. **Supplementary Table S7.** Covariate testing for maternal age. **Supplementary Table S8.** sensitivity analyses for a group without comorbid diagnoses. **Supplementary Table S9.** Comparison of risk factors in the total SM sample and in SM cases diagnosed since 1998. **Supplementary Table S10.** Sensitivity analyses for a group without parental diagnoses before 1998. **Supplementary Table S11.** Sensitivity analyses for a group without parental diagnoses before 1998 and without cases diagnosed only before 1998.

## Data Availability

The data in the present article is based on Finnish national health registers and cannot be shared online. All the material and tables used in the paper are included in the paper or provided as supplementary tables.

## Abbreviations

SM: Selective mutism
ICD-10: International Classification of Diseases, Tenth Revision
ICD-9: International Classification of Diseases, Ninth Revision
DSM-5: Diagnostic and Statistical Manual of Mental Disorders
SES: Socioeconomic status
FHDR: The Finnish Hospital Discharge Register
FIPS-Anxiety: The Finnish Prenatal Study of Anxiety disorders
FCPR: The Finnish Central Population Register
FMBR: The Finnish Medical Birth Register
PIC: Personal identity code
ADHD: Attention-deficit/hyperactivity disorder
ASD: Autism spectrum disorders
OR: Odds ratio
CI: Confidence interval
