# Supplementary material for: The impact of parental psychopathology and sociodemographic factors in selective mutism - a nationwide population-based study
Source: BMC Psychiatry. 2020 May 12;20:221. doi: 10.1186/s12888-020-02637-6 (PMC7216734; doi:10.1186/s12888-020-02637-6)
Supplement: Supplementary file 1 — Additional file 1: Supplementary Table S1. Inclusion and exclusion criteria of Selective Mutism. Supplementary Table S2. Diagnostic categories and codes for parental psychiatric disorders. Supplementary Table S3. Diagnostic categories and codes for comorbid psychiatric disorders in children. Supplementary Table S4. Covariate testing for maternal psychopathology. Supplementary Table S5. Covariate testing for parental psychopathology. Supplementary Table S6. Covariate testing for paternal age. Supplementary Table S7. Covariate testing for maternal age. Supplementary Table S8. sensitivity analyses for a group without comorbid diagnoses. Supplementary Table S9. Comparison of risk factors in the total SM sample and in SM cases diagnosed since 1998. Supplementary Table S10. Sensitivity analyses for a group without parental diagnoses before 1998. Supplementary Table S11. Sensitivity analyses for a group without parental diagnoses before 1998 and without cases diagnosed only before 1998. [file 12888_2020_2637_MOESM1_ESM.docx]

**Supplementary table S1** Inclusion and exclusion criteria of Selective Mutism

Matched controls from FMBR at a 1:4 ratio N=4528

Cases identified from FHDR N=1114

143 controls with anxiety or childhood emotional disorders excluded

42 children diagnosed after age 15 excluded with their matched controls.

860 cases and 3250 controls included in the final analyses

36 controls with ASD and 2 controls with moderate or severe intellectual disability and 2 controls with both ASD and intellectual disability excluded

203 cases with coexisting ASD, 3 cases with comorbid moderate/severe intellectual disability and ASD, and 6 cases with comorbid psychotic disorder diagnosed before or at the same time with SM excluded along with their controls.

**Supplementary table S2** Diagnostic categories and codes for parental psychiatric disorders

|  | **ICD-10** | **ICD-9** | **ICD-8** |
| --- | --- | --- | --- |
| **Schizophrenia and schizoaffective** | F20, F25 | 295 | 295 |
| **Selective mutism** | F94.0 | 3132C | - |
| **Other Psychoses (excluding schizophrenia and schizoaffective)** | F21-24, F28, F29 | 297, 2989X, 3012C | 297, 298.20, 298.30, 298.99, 299 |
| **Bipolar disorder** | F30, F31 | 2962A-G, 2963A-G, 2964A-G, 2967A | 296.10, 296.30, 298.10 |
| **Unipolar mood disorders** | F32, F33, F34, F38, F39 | 2961A-G, 2968A, 3004A, 2988A | 296 (excluding 296.10 and 296.30), 298.00, 298.10, 300.41 |
| **Anxiety disorders (including Childhood anxiety disorders)** | F40-42 (excluding F41.2), F93 | 3000A, 3000B, 3000C, 3002B, 3002C, 3002D, 3002X, 3003A, 3092A, 3092B, 3133A | 300.00, 300.20, 300.30 |
| **Personality disorders** | F60-62 | 301 (excluding 3012C) | 301 |
| **Alcohol and drug addiction/abuse** | F10-19 | 303–305, 291–292 | 303–304, 291, 294.30 |
| **ADHD** | F90 | 314 |  |
| **Autism spectrum disorders** | F84 | 299 |  |
| **Conduct and oppositional disorders** | F90.1, F91, F92 | 3120A, 3123D, 3138A | 308.99 |
| **Learning and coordination disorders** | F80-83 | 315 | 306.00, 306.10, 306.30 |
| **Intellectual disability** | F70–F79 | 317-319 | 310-315 |
| **Other psychiatric disorders** | F41.2, F43-45, F48, F50-55, F59, F63-66, F68-69, F88-89, F94-95 (excluding F94.0), F98, F99 | 300 (excluding 3000A, 3000B, 3000C, 3002B, 3002C, 3002D, 3002X, 3003A, 3004A and 3012C), 302, 3071A, 3074A, 3074F, 3074H, 3075A-C, 3075E, 3078A, 3079X, 309 (excluding 3092A, 3092B), 312 (excluding 3120A and 3123D) | 300 (excluding 300.00, 300.20, 300.30, 300.41), 302, 305, 306.20, 306.40, 306.50, 306.60, 306.70, 306.98, 307.99 |

**Supplementary table S3** Diagnostic categories and codes for comorbid psychiatric disorders in children

|  | **ICD-10** | **ICD-9** |
| --- | --- | --- |
| **1.Any psychiatric or neurodevelopmental disorder** | F10-F99 | 291-319, excluding 316 |
| a. Schizophrenia spectrum disorders | F20 schizophrenia, F21  schizotypal disorder, F22  delusional disorder, F23 acute  polymorphic psychotic disorder without symptoms of  schizophrenia, F24 induced  delusional disorder, F25  schizoaffective disorder, F28  other nonorganic psychotic  disorders, F29 unspecified  nonorganic disorders | 295, 297, 2989X, 3012C |
| b. Affective disorders | F30 hypomania, F31 bipolar  affective disorder, F32  depressive episode, F33  recurrent depressive episode,  F34 cyclothymia and dysthymia,  F38 other single mood  (affective) disorder, F39  unspecified mood (affective)  disorder | 296, 3004A, 2988A |
| *Bipolar disorders* | *F30, F31* | *2962A-G, 2963A-G, 2964A-G, 2967A* |
| *Unipolar disorders* | *F32, F33, F34, F38, F39* | *2961A-G, 2968A, 3004A, 2988A* |
| c. Anxiety disorders | F40 phobic anxiety disorders,  F41 other anxiety disorders  (excluding F41.2), F42  obsessive– compulsive disorder | 3000A, 3000B, 3000C, 3002B,  3002C, 3002D, 3002X, 3003A |
| *Phobic anxiety disorders* | *F40* | *3002X* |
| *Social phobia* | *F40.1* | *3002D* |
| *Other anxiety disorders* | *F41 (excluding F41.2)* | *3000A,B,C, 3002B, 3002C* |
| *Obsessive– compulsive disorders* | *F42* | *3003A* |
| d. Other neurotic and personality disorders | F41.2 mixed anxiety and  depression, F43 reaction to  severe stress and adjustment  disorders, F44 dissociative  amnesia, F45 somatoform  disorders, F48 other neurotic  disorders, F50 eating disorders,  F51 non-organic sleep disorders  (excluding F51.3; F51.4), F52  lack or loss of sexual desire,  F53 mental and behavioral  disorders associated with the  puerperium, not elsewhere  classified, F54 psychological  and behavioral factors  associated with disorders or  diseases classified elsewhere,  F55 abuse of non-dependence  producing substances, F59  unspecified behavioral  syndromes associated with  physiological disturbances, F60  specific personality disorders,  F61 mixed and other personality disorders, F62 enduring  personality changes, not  attributable to brain damage and diseases, F63 habit and impulse disorders, F64 gender identity disorders, F65 fetishism, F66 psychological and behavioural disorders associated with sexual  development and orientation,  F68 other disorders of adult  personality and behavior, F69  unspecified disorder of adult  personality and behavior, F99  mental disorder not otherwise  specified | 300–302 (excluding 3000A,  3000B, 3000C, 3002B, 3002C,  3002D, 3002X, 3003A, 3004A and  3012C), 3071A, 3074A, 3074F,  3074H, 3075A, 3075B, 3075C,  3075E, 3078A, 3079X, 309  (excluding 3092A and 3092B), 312 (excluding 3120A, 3123C and  3123D) |
| e. Substance abuse disorder | Mental and behavioural  disorders due to use of… F10  alcohol, F11 opioids, F12  cannabinoids, F13 sedatives or  hypnotics, F14 cocaine, F15  other stimulants, including  caffeine, F16 hallucinogens,  F17 tobacco, F18 volatile  solvents, F19 multiple drug use  and use of other psychoactive  substances | 303-305, 291-292 |
| a. Attention deficit hyperactivity disorder | F90 hyperkinetic disorders | 314 |
| b. Intellectual disability* | F70-F79 intellectual disability | 317-319 |
| c. Childhood emotional disorders | F93 emotional disorders with  onset specific to childhood | 3092A, 3092B, 3133A |
| *Separation anxiety* | *F93.0* | *3092A* |
| *Childhood onset phobic- and anxiety disorders* | *F93.1* | *3092B* |
| *Childhood onset social phobia* | *F93.2* |  |
| *Other childhood onset emotional and anxiety disorders* | *F93.3-9* | *3138X, 3133A* |
| d. Conduct and oppositional disorders | F91-F92 conduct disorders,  including oppositional defiant  disorder | 3120A, 3123C, 3123D, 3138A |
| e. Tic disorders | F95 tic disorders | 3072A, 3072B, 3072C, 3072D |
| f. Learning and coordination disorders | F80-F83 learning disabilities or  motor coordination disorders,  including developmental  disorders of speech, language,  scholastic skills or motor  coordination | 315 |
| *Specific developmental disorders of speech and language* | *F80* |  |
| *Specific developmental disorder of scholastic skills* | *F81* |  |
| *Specific developmental disorder of motor function* | *F82* |  |
| *Mixed specific developmental disorder* | *F83* |  |

**Supplementary table S4** Covariate testing for maternal psychopathology

|  | Maternal psychopathology | | | Relation between covariates and outcome |
| --- | --- | --- | --- | --- |
| Covariates | **Yes** | **No** | **P-value** | **P-value** |
| Maternal age |  |  | <0.0001 | 0.0012 |
| <20 | 29 (5.7) | 60 (2.2) |  |  |
| 20-24 | 107 (21.1) | 436 (15.9) |  |  |
| 25-29 | 150 (29.5) | 964 (35.2) |  |  |
| 30-34 | 138 (27.2) | 831 (30.3) |  |  |
| 35-39 | 67 (13.2) | 375 (13.7) |  |  |
| ≥40 | 17 (3.4) | 76 (2.8) |  |  |
| Paternal age |  |  | 0.0006 | 0.0001 |
| <20 | 8 (1.6) | 13 (0.5) |  |  |
| 20-24 | 72 (14.4) | 263 (9.7) |  |  |
| 25-29 | 129 (25.8) | 745 (27.4) |  |  |
| 30-34 | 150 (30.0) | 953 (35.1) |  |  |
| 35-39 | 87 (17.4) | 491 (18.1) |  |  |
| ≥40 | 54 (10.8) | 254 (9.3) |  |  |
| Maternal socioeconomic status |  |  | 0.0006 | <0.0001 |
| Upper white-collar worker | 56 (11.0) | 439 (16.0) |  |  |
| Lower white-collar worker | 171 (33.7) | 1012 (36.9) |  |  |
| Blue-collar worker | 77 (15.2) | 441 (16.1) |  |  |
| Other | 101 (19.9) | 407 (14.8) |  |  |
| Missing | 103 (20.3) | 443 (16.2) |  |  |
| Marital status^a^ |  |  | 0.197 | <0.0001 |
| Married/in a relationship | 446 (95.9) | 2465 (97.1) |  |  |
| Single | 19 (4.1) | 75 (3.0) |  |  |
| Immigration status |  |  | 0.723 | 0.306 |
| Both parents Finnish | 484 (95.3) | 2586 (94.3) |  |  |
| Mother immigrant | 8 (1.6) | 42 (1.5) |  |  |
| Father immigrant | 9 (1.8) | 57 (2.1) |  |  |
| Both parents immigrants | 7 (1.4) | 57 (2.1) |  |  |
| Urbanicity^b^ |  |  |  |  |
| Urban | 318 (62.6) | 1652 (60.9) | 0.595 | 0.351 |
| Semi-urban | 88 (17.3) | 462 (17.0) |  |  |
| Rural | 102 (20.1) | 600 (22.1) |  |  |
| Paternal psychopathology^c^ | 108 (21.6) | 294 (10.8) | <0.0001 | <0.0001 |

^a^Missing information: 63 (7.3%) cases, 245 (7.5%) controls

^b^Missing information: 3 (0.4%) cases, 28 controls (0.9%)

^c^Missing information: 19 cases (2.2%), 31 controls (1.0%)

**Supplementary table S5** Covariate testing for parental psychopathology

|  | Paternal psychopathology | | | Relation between covariates and outcome |
| --- | --- | --- | --- | --- |
| Covariates | **Yes** | **No** | **P-value** | **P-value** |
| Maternal age |  |  | <0.0001 | 0.0012 |
| <20 | 27 (6.7) | 58 (2.1) |  |  |
| 20-24 | 108 (26.9) | 431 (15.3) |  |  |
| 25-29 | 101 (25.1) | 1008 (35.8) |  |  |
| 30-34 | 115 (28.6) | 842 (29.9) |  |  |
| 35-39 | 41 (10.2) | 398 (14.1) |  |  |
| ≥40 | 10 (2.5) | 80 (2.8) |  |  |
| Paternal age |  |  | <0.0001 | 0.0001 |
| <20 | 8 (2.0) | 13 (0.5) |  |  |
| 20-24 | 73 (18.2) | 262 (9.3) |  |  |
| 25-29 | 99 (24.6) | 775 (27.5) |  |  |
| 30-34 | 120 (29.9) | 983 (34.9) |  |  |
| 35-39 | 66 (16.4) | 512 (18.2) |  |  |
| ≥40 | 36 (9.0) | 272 (9.7) |  |  |
| Maternal socioeconomic status |  |  | 0.0012 | <0.0001 |
| Upper white-collar worker | 43 (10.7) | 450 (16.0) |  |  |
| Lower white-collar worker | 130 (32.3) | 1046 (37.1) |  |  |
| Blue-collar worker | 81 (20.2) | 428 (15.2) |  |  |
| Other | 77 (19.2) | 426 (15.1) |  |  |
| Missing | 71 (17.7) | 467 (16.6) |  |  |
| Marital status^a^ |  |  | <0.0001 | <0.0001 |
| Married/in a relationship | 343 (93.2) | 2560 (97.8) |  |  |
| Single | 25 (6.8) | 58 (2.2) |  |  |
| Immigration status |  |  | 0.644 | 0.306 |
| Both parents Finnish | 384 (95.5) | 2655 (94.3) |  |  |
| Mother immigrant | 6 (1.5) | 44 (1.6) |  |  |
| Father immigrant | 5 (1.2) | 61 (2.2) |  |  |
| Both parents immigrants | 7 (1.7) | 57 (2.0) |  |  |
| Urbanicity^b^ |  |  | 0.204 | 0.351 |
| Urban | 238 (59.4) | 1712 (61.3) |  |  |
| Semi-urban | 81 (20.2) | 466 (16.7) |  |  |
| Rural | 82 (20.5) | 617 (22.1) |  |  |
| Maternal psychopathology | 108 (26.9) | 392 (13.9) | <0.0001 | <0.0001 |

Missing paternal information: 19 cases (2.2%), 31 controls (1.0%)

^a^Missing information: 63 (7.3%) cases, 245 (7.5%) controls

^b^Missing information: 3 (0.3%) cases, 28 controls (0.9%)

**Supplementary table S6** Covariate testing for paternal age.

|  | Paternal age | | | | | | | Relation between covariates and outcome P-value |
| --- | --- | --- | --- | --- | --- | --- | --- | --- |
| Covariates | **<20 years N (%)** | **20-24 years N (%)** | **25-29 years N (%)** | **30-34 years N (%)** | **35-39 years**  **N (%)** | **≥40 years**  **N (%)** | **P-value** |  |
| Maternal age |  |  |  |  |  |  | <0.0001 | 0.0012 |
| <20 | 16 (76.2) | 50 (14.9) | 14 (1.6) | 4 (1.6) | 0 (0.0) | 1 (0.3) |  |  |
| 20-24 | 4 (19.1) | 226 (67.5) | 222 (25.4) | 63 (5.7) | 13 (2.3) | 11 (3.6) |  |  |
| 25-29 | 1 (4.8) | 53 (15.8) | 506 (57.9) | 422 (38.3) | 99 (17.1) | 28 (9.1) |  |  |
| 30-34 | 0 (0.0) | 5 (1.5) | 118 (13.5) | 522 (47.3) | 233 (40.3) | 79 (25.7) |  |  |
| 35-39 | 0 (0.0) | 0 (0.0) | 14 (1.6) | 85 (7.7) | 209 (36.2) | 131 (42.5) |  |  |
| ≥40 | 0 (0.0) | 1 (0.3) | 0 (0.0) | 7 (0.6) | 24 (4.2) | 58 (18.8) |  |  |
| Maternal socioeconomic status |  |  |  |  |  |  | <0.0001 | <0.0001 |
| Upper white-collar worker | 0 (0.0) | 6 (1.8) | 84 (9.6) | 196 (17.8) | 132 (22.8) | 75 (24.4) |  |  |
| Lower white-collar worker | 3 (14.3) | 81 (24.2) | 311 (35.6) | 459 (41.6) | 228 (39.5) | 94 (30.5) |  |  |
| Blue-collar worker | 2 (9.5) | 76 (22.7) | 175 (19.9) | 136 (12.3) | 68 (11.8) | 53 (17.2) |  |  |
| Other | 9 (42.9) | 110 (32.8) | 151 (17.3) | 120 (10.9) | 72 (12.5) | 41 (13.3) |  |  |
| Missing | 7 (33.3) | 62 (18.5) | 154 (17.6) | 192 (17.4) | 78 (13.5) | 45 (14.6) |  |  |
| Marital status^a^ |  |  |  |  |  |  | <0.0001 | <0.0001 |
| Married/in a relationship | 12 (70.6) | 280 (94.0) | 788 (97.8) | 1011 (98.3) | 531 (97.4) | 281 (96.6) |  |  |
| Single | 5 (29.4) | 18 (6.0) | 18 (2.2) | 18 (1.8) | 14 (2.6) | 10 (3.4) |  |  |
| Immigration |  |  |  |  |  |  | 0.0002 | 0.306 |
| Both parents Finnish | 21 (100.0) | 321 (95.8) | 838 (95.9) | 1046 (94.8) | 539 (93.3) | 274 (89.0) |  |  |
| Mother immigrant | 0 (0.0) | 0 (0.0) | 9 (1.0) | 19 (1.7) | 7 (1.2) | 15 (4.9) |  |  |
| Father immigrant | 0 (0.0) | 7 (2.1) | 14 (1.6) | 22 (2.0) | 14 (2.4) | 9 (2.9) |  |  |
| Both parents immigrants | 0 (0.0) | 7 (2.1) | 13 (1.5) | 16 (1.5) | 18 (3.1) | 10 (3.3) |  |  |
| Urbanicity^b^ |  |  |  |  |  |  | 0.618 | 0.351 |
| Urban | 12 (57.1) | 29 (62.6) | 524 (60.2) | 697 (63.7) | 330 (57.8) | 178 (58.2) |  |  |
| Semi-urban | 4 (19.1) | 57 (17.1) | 153 (17.6) | 169 (15.5) | 106 (18.6) | 58 (18.9) |  |  |
| Rural | 5 (23.8) | 68 (20.4) | 193 (22.2) | 228 (20.8) | 135 (23.6) | 70 (22.9) |  |  |
| Maternal psychopathology | 8 (38.1) | 72 (21.5) | 129 (14.8) | 150 (13.6) | 87 (15.1) | 54 (17.5) | 0.0006 | <0.0001 |
| Paternal psychopathology^c^ | 8 (38.1) | 73 (21.8) | 99 (11.3) | 120 (10.9) | 66 (11.4) | 36 (11.7) | <0.0001 | <0.0001 |

^a^Missing information: 63 (7.3%) cases, 245 (7.5%) controls

^b^Missing information: 3 (0.4%) cases, 28 controls (0.9%)

^c^Missing information: 19 cases (2.2%), 31 controls (1.0%)

**Supplementary table S7** Covariate testing for maternal age.

|  | Maternal age | | | | | | | Relation between covariates and outcome P-value |
| --- | --- | --- | --- | --- | --- | --- | --- | --- |
| Covariates | **<20 years N (%)** | **20-24 years N (%)** | **25-29 years N (%)** | **30-34 years N (%)** | **35-39 years**  **N (%)** | **≥40 years**  **N (%)** | **P-value** |  |
| Paternal age |  |  |  |  |  |  | <0.0001 | 0.0001 |
| <20 years | 16 (18.8) | 4 (0.7) | 1 (0.1) | 0 (0.0) | 0 (0.0) | 0 (0.0) |  |  |
| 20-24 years | 50 (58.8) | 226 (41.9) | 53 (4.8) | 5 (0.5) | 0 (0.0) | 1 (1.1) |  |  |
| 25-29 years | 14 (16.5) | 222 (41.2) | 506 (45.6) | 118 (12.3) | 14 (3.2) | 0 (0.0) |  |  |
| 30-34 years | 4 (4.7) | 63 (11.7) | 422 (38.1) | 522 (54.6) | 85 (19.4) | 7 (7.8) |  |  |
| 35-39 years | 0 (0.0) | 13 (2.4) | 99 (8.9) | 233 (24.4) | 209 (47.6) | 24 (26.7) |  |  |
| ≥40years | 1 (1.2) | 11 (2.0) | 28 (2.5) | 79 (8.3) | 131 (29.8) | 58 (64.4) |  |  |
| Maternal socioeconomic status |  |  |  |  |  |  | <0.0001 | <0.0001 |
| Upper white-collar worker | 0 (0.0) | 8 (1.5) | 126 (11.3) | 220 (22.7) | 121 (27.4) | 20 (21.5) |  |  |
| Lower white-collar worker | 12 (13.5) | 149 (27.4) | 436 (39.1) | 388 (40.0) | 169 (38.2) | 29 (31.2) |  |  |
| Blue-collar worker | 10 (11.2) | 128 (23.6) | 188 (16.9) | 123 (12.7) | 55 (12.4) | 14 (15.1) |  |  |
| Other | 41 (46.1) | 156 (28.7) | 154 (13.8) | 99 (10.2) | 44 (10.0) | 14 (15.1) |  |  |
| Missing | 26 (29.2) | 102 (18.7) | 210 (18.7) | 139 (14.3) | 53 (11.9) | 16 (17.2) |  |  |
| Marital status^a^ |  |  |  |  |  |  | <0.0001 | <0.0001 |
| Married/in a relationship | 64 (85.3) | 472 (95.9) | 1008 (98.0) | 877 (96.9) | 407 (97.4) | 83 (96.5) |  |  |
| Single | 11 (14.7) | 20 (4.1) | 21 (2.0) | 28 (3.1) | 11 (2.6) | 3 (3.5) |  |  |
| Immigration status |  |  |  |  |  |  | 0.763 | 0.306 |
| Both parents Finnish | 87 (97.8) | 508 (93.6) | 1059 (95.1) | 914 (94.3) | 413 (93.4) | 89 (95.7) |  |  |
| Mother immigrant | 1 (1.1) | 7 (1.3) | 17 (1.5) | 18 (1.9) | 6 (1.4) | 1 (1.1) |  |  |
| Father immigrant | 0 (0.0) | 12 (2.2) | 18 (1.6) | 20 (2.1) | 14 (3.2) | 2 (2.2) |  |  |
| Both parents immigrants | 1 (1.1) | 16 (3.0) | 20 (1.8) | 17 (1.8) | 9 (2.0) | 1 (1.1) |  |  |
| Urbanicity^b^ |  |  |  |  |  |  | 0.760 | 0.351 |
| Urban | 54 (60.7) | 322 (59.4) | 676 (61.1) | 590 (61.8) | 268 (61.3) | 60 (65.9) |  |  |
| Semi-urban | 20 (22.5) | 87 (16.1) | 195 (17.6) | 158 (16.5) | 75 (17.1) | 15 (16.5) |  |  |
| Rural | 15 (16.9) | 133 (24.5) | 236 (21.3) | 207 (21.7) | 95 (21.7) | 16 (17.6) |  |  |
| Maternal psychopathology | 29 (32.6) | 107 (19.7) | 150 (13.5) | 138 (14.2) | 67 (15.2) | 17 (18.3) | <0.0001 | <0.0001 |
| Paternal psychopathology^c^ | 27 (31.8) | 108 (20.0) | 101 (9.1) | 115 (12.0) | 41 (9.3) | 10 (11.1) | <0.0001 | <0.0001 |

^a^Missing information: 63 (7.3%) cases, 245 (7.5%) controls

^b^Missing information: 3 (0.4%) cases, 28 controls (0.9%)

^c^Missing information: 19 cases (2.2%), 31 controls (1.0%)

**Supplementary table S8:** sensitivity analyses for a group without comorbid diagnoses.

|  | ***Total SM)***  ***(n=860)*** | ***Matched controls (n=3,250)*** | ***SM without comorbid diagnoses (n=266)*** | ***Matched controls (n=1006)*** | ***P-value**** |
| --- | --- | --- | --- | --- | --- |
| ***PSYCHOPATHOLOGY*** |  |  |  |  | *0.0591* |
| *Only mothers* | *163 (19.4)* | *392 (12.2)* | *43 (16.4)* | *117 (11.8)* |  |
| *Only fathers* | *108 (12.8)* | *294 (9.1)* | *24 (9.2)* | *91 (9.1)* |  |
| *Both* | *69 (8.2)* | *108 (3.4)* | *15 (5.7)* | *32 (3.2)* |  |
| *None* | *501 (59.6)* | *2,425 (75.3)* | *180 (68.7)* | *756 (75.9)* |  |
| ***MATERNAL SES*** | *n=860* | *n=3,250* | *n=266* | *n=1006* | *0.8999* |
| *Lower white collar workers* | 296 (34.4) | *1,183 (36.4)* | *93 (35.0)* | *370 (36.8)* |  |
| *Blue collar workers* | *188 (21.9)* | *518 (15.9)* | *52 (19.6)* | *161 (16.0)* |  |
| *Others* | *166 (19.3)* | *508 (15.6)* | *50 (18.8)* | *138 (13.7)* |  |
| *Missing* | *143 (16.6)* | *546 (16.8)* | *47 (17.7)* | *187 (18.6)* |  |
| *Upper white collar workers* | *67 (7.8)* | *495 (15.2)* | *24 (9.0)* | *150 (14.9)* |  |
| ***MARITAL STATUS*** | *n=797* | *n=3005* | *n=246* | *n=928* | *0.1119* |
| *Single* | *55 (6.9)* | *94 (3.1)* | *10 (4.1)* | *36 (3.9)* |  |
| *Married/in a relationship* | *742 (93.1)* | *2911 (96.9)* | *236 (95.9)* | *892 (96.1)* |  |
| ***RESIDENCE*** | *n=857* | *n=3,222* | *n=266* | *n=996* | *0.3513* |
| *Urban* | *523 (61.0)* | *1,970 (61.1)* | *150 (56.4)* | *582 (58.4)* |  |
| *Semi-urban* | *132 (15.4)* | *550 (17.1)* | *49 (18.4)* | *185 (18.6)* |  |
| *Rural* | *202 (23.6)* | *702 (21.8)* | *67 (25.2)* | *229 (23.0)* |  |
| ***MATERNAL AGE*** | *n=860* | *n=3,250* | *n=266* | *n=1006* | *0.4519* |
| *≤19* | *27 (3.1)* | *89 (2.7)* | *6 (2.3)* | *29 (2.9)* |  |
| *20-24* | *174 (20.2)* | *543 (16.7)* | *61 (22.9)* | *176 (17.5)* |  |
| *25-29* | *261 (30.4)* | *1114 (34.3)* | *66 (24.8)* | *339 (33.7)* |  |
| *30-34* | *222 (25.8)* | *969 (29.8)* | *78 (29.3)* | *292 (29.0)* |  |
| *35-39* | *137 (15.9)* | *442 (13.6)* | *45 (16.9)* | *140 (13.9)* |  |
| *≥40* | *39 (4.5)* | *93 (2.9)* | *10 (3.8)* | *30 (3.0)* |  |
| ***PATERNAL AGE*** | *n=841* | *n=3,219* | *n=262* | *n=996* | *0.7667* |
| *≤19* | *7 (0.8)* | *21 (0.7)* | *1 (0.4)* | *7 (0.7)* |  |
| *20-24* | *85 (10.2)* | *335 (10.4)* | *23 (8.8)* | *120 (12.1)* |  |
| *25-29* | *210 (25.0)* | *874 (27.2)* | *60 (22.9)* | *256 (25.7)* |  |
| *30-34* | *238 (28.3)* | *1,103 (34.3)* | *80 (30.5)* | *332 (33.3)* |  |
| *35-39* | *178 (21.2)* | *578 (18.0)* | *63 (24.1)* | *189 (19.0)* |  |
| *≥40* | *123 (14.6)* | *308 (9.6)* | *35 (13.4)* | *92 (9.2)* |  |
| I**MMIGRATION** | *n=860* | *n=3250* |  |  | *0.9669* |
| Both parents Finnish | *815 (94.8)* | *3070 (94.5)* | *250 (94.0)* | *946 (94.0)* |  |
| Mother immigrant | *19 (2.2)* | *50 (1.5)* | *7 (2.6)* | *16 (1.6)* |  |
| Father immigrant | *12 (1.4)* | *66 (2.0)* | *4 (1.5)* | *24 (2.4)* |  |
| Both parents immigrants | *14 (1.6)* | *64 (2.0)* | *5 (1.9)* | *20 (2.0)* |  |

In groups with P-value over 0.05, there is no statistically significant difference between original data and the sub-group.

**Supplementary table S9**: Comparison of risk factors in the total SM sample and in SM cases diagnosed since 1998

|  | ***All SM cases***  ***(n=860)*** | ***Matched controls (n=3,250)*** | ***P-value*** | ***SM diagnosed since 1998 (n=842)*** | ***Matched controls (n=3,180)*** | ***P-value*** |
| --- | --- | --- | --- | --- | --- | --- |
| **PSYCHOPATHOLOGY** | *n=841* | *n=3219* | ***P=<0.0001*** | *n=823* | *n=3149* | ***P=<0.0001*** |
| *Only mothers* | *163 (19.4)* | *392 (12.2)* |  | *158 (19.2)* | *382 (12.1)* |  |
| *Only fathers* | *108 (12.8)* | *294 (9.1)* |  | *106 (12.9)* | *286 (9.1)* |  |
| *Both* | *69 (8.2)* | *108 (3.4)* |  | *66 (8.0)* | *105 (3.3)* |  |
| *None* | *501 (59.6)* | *2,425 (75.3)* |  | *493 (59.9)* | *2376 (75.5)* |  |
| **MATERNAL SES** | *n=860* | *n=3,250* | ***P=<0.0001*** | *n=842* | *n=3180* | ***P=<0.0001*** |
| *Lower white collar workers* | 296 (34.4) | *1,183 (36.4)* |  | *293 (34.8)* | *1172 (36.9)* |  |
| *Blue collar workers* | *188 (21.9)* | *518 (15.9)* |  | *188 (22.3)* | *512 (16.1)* |  |
| *Others* | *166 (19.3)* | *508 (15.6)* |  | *163 (19.4)* | *507 (15.9)* |  |
| *Missing* | *143 (16.6)* | *546 (16.8)* |  | *132 (15.7)* | *497 (15.6)* |  |
| *Upper white collar workers* | *67 (7.8)* | *495 (15.2)* |  | *66 (7.8)* | *492 (15.5)* |  |
| **MARITAL STATUS** | *n=797* | *n=3005* | ***P=<0.0001*** | *n=780* | *n=2939* | ***P=<0.0001*** |
| *Single* | *55 (6.9)* | *94 (3.1)* |  | *55 (7.1)* | *94 (3.2)* |  |
| *Married/in a relationship* | *742 (93.1)* | *2911 (96.9)* |  | *725 (93.0)* | *2845 (96.8)* |  |
| **RESIDENCE** | *n=857* | *n=3,222* | ***P=0.3535*** | *n=839* | *n=3152* | ***P=0.4347*** |
| *Urban* | *523 (61.0)* | *1,970 (61.1)* |  | *514 (61.3)* | *1923 (61.0)* |  |
| *Semi-urban* | *132 (15.4)* | *550 (17.1)* |  | *130 (15.5)* | *540 (17.1)* |  |
| *Rural* | *202 (23.6)* | *702 (21.8)* |  | *195 (23.2)* | *689 (21.9)* |  |
| **MATERNAL AGE** | *n=860* | *n=3,250* | ***P=0.0010*** | *n=842* | *n=3180* | ***P=0.0013*** |
| *≤19* | *27 (3.1)* | *89 (2.7)* |  | *27 (3.2)* | *87 (2.7)* |  |
| *20-24* | *174 (20.2)* | *543 (16.7)* |  | *169 (20.1)* | *533 (16.8)* |  |
| *25-29* | *261 (30.4)* | *1114 (34.3)* |  | *254 (30.2)* | *1084 (34.1)* |  |
| *30-34* | *222 (25.8)* | *969 (29.8)* |  | *217 (25.8)* | *947 (29.8)* |  |
| *35-39* | *137 (15.9)* | *442 (13.6)* |  | *136 (16.2)* | *436 (13.7)* |  |
| *≥40* | *39 (4.5)* | *93 (2.9)* |  | *39 (4.6)* | *93 (2.9)* |  |
| **PATERNAL AGE** | *n=841* | *n=3,219* | ***P=0.0001*** | *n=823* | *n=3149* | ***P=<0.0001*** |
| *≤19* | *7 (0.8)* | *21 (0.7)* |  | *7 (0.9)* | *20 (0.6)* |  |
| *20-24* | *85 (10.2)* | *335 (10.4)* |  | *81 (9.8)* | *328 (10.4)* |  |
| *25-29* | *210 (25.0)* | *874 (27.2)* |  | *205 (24.9)* | *851 (27.0)* |  |
| *30-34* | *238 (28.3)* | *1,103 (34.3)* |  | *234 (28.4)* | *1078 (34.2)* |  |
| *35-39* | *178 (21.2)* | *578 (18.0)* |  | *175 (21.3)* | *571 (18.1)* |  |
| *≥40* | *123 (14.6)* | *308 (9.6)* |  | *121 (14.7)* | *301 (9.6)* |  |
| I**MMIGRATION** | *n=860* | *n=3250* | ***P=0.3088*** | *n=842* | *n=3180* | ***P=0.3088*** |
| *Both parents Finnish* | *815 (94.8)* | *3070 (94.5)* |  | *797 (94.7)* | *3000 (94.3)* |  |
| *Mother immigrant* | *19 (2.2)* | *50 (1.5)* |  | *19 (2.3)* | *50 (1.6)* |  |
| *Father immigrant* | *12 (1.4)* | *66 (2.0)* |  | *12 (1.4)* | *66 (2.1)* |  |
| *Both parents immigrants* | *14 (1.6)* | *64 (2.0)* |  | *14 (1.7)* | *64 (2.0)* |  |

**Supplementary table S10:** Sensitivity analyses for a group without parental diagnoses before 1998

|  | **Mothers** | | | **Fathers** | | |
| --- | --- | --- | --- | --- | --- | --- |
|  | **Cases (N=860)**  **N (%)** | **Controls (N=3,250)**  **N (%)** | **P-value** | **Cases (N=841)**  **N (%)** | **Controls (N=3,219)**  **N (%)** | **P-value** |
| **Schizophrenia and**  **schizoaffective disorders** | 17 (2.0) | 10 (0.3) | **P=<0.0001** | 7 (0.8) | 8 (0.3) | **P=0.0258** |
| **Selective mutism** | 0 (0.0) | 0 (0.0) | **N/A** | 0 (0.0) | 0 (0.0) | **N/A** |
| **Other psychoses** | 29 (3.4) | 23 (0.7) | **P=<0.0001** | 12 (1.4) | 19 (0.6) | **P=0.0065** |
| **Bipolar disorders** | 17 (2.0) | 24 (0.7) | **P=0.0017** | 14 (1.7) | 35 (1.1) | **P=0.1469** |
| **Unipolar mood disorders** | 154 (17.9) | 269 (8.3) | **P=<0.0001** | 61 (7.3) | 134 (4.2) | **P=0.0002** |
| **Anxiety disorders (including childhood anxiety disorders)** | 57 (6.6) | 105 (3.2) | **P=<0.0001** | 24 (2.9) | 60 (1.9) | P=0.0705 |
| **Personality disorders** | 42 (4.9) | 47 (1.5) | **P=<0.0001** | 26 (3.1) | 50 (1.6) | **P=0.0039** |
| **Alcohol and drug addiction/abuse** | 41 (4.8) | 77 (2.4) | **P=0.0003** | 73 (8.7) | 126 (3.9) | **P=<0.0001** |
| **Attention-deficit/**  **hyperactivity disorders** | 3 (0.4) | 11 (0.3) | **P=0.9474** | 3 (0.4) | 4 (0.1) | **P=0.1503** |
| **Autism spectrum disorders** | 0 (0.0) | 1 (0.03) | **N/A** | 3 (0.4) | 1 (0.03) | **N/A** |
| **Conduct/oppositional defiant disorders** | 1 (0.1) | 1 (0.03) | **N/A** | 0 (0.0) | 2 (0.1) | **N/A** |
| **Learning and**  **coordination disorders** | 4 (0.5) | 2 (0.1) | **P=0.019** | 3 (0.4) | 1 (0.03) | **P=0.0314** |
| **Intellectual disability** | 2 (0.2) | 0 (0.0) | **N/A** | 0 (0.0) | 1 (0.03) | **N/A** |
| **Other psychiatric disorders** | 84 (9.8) | 228 (7.0) | **P=0.0056** | 37 (4.4) | 114 (3.5) | **P=0.2858** |

P-values in groups, where significance remains similar in the whole data and sub-data are **bolded**

**Supplementary table S11**: Sensitivity analyses for a group without parental diagnoses before 1998 and without cases diagnosed only before 1998

|  | **Mothers** | | | **Fathers** | | |
| --- | --- | --- | --- | --- | --- | --- |
|  | **Cases (N=842)**  **N (%)** | **Controls (N=3,180)**  **N (%)** | **P-value** | **Cases (N=823)**  **N (%)** | **Controls (N=3,149)**  **N (%)** | **P-value** |
| **Schizophrenia and**  **schizoaffective disorders** | 17 (2.0) | 9 (0.3) | **P=<0.0001** | 7 (0.9) | 8 (0.3) | **P=0.0258** |
| **Selective mutism** | 0 (0.0) | 0 (0.0) | **N/A** | 0 (0.0) | 0 (0.0) | **N/A** |
| **Other psychoses** | 28 (3.3) | 21 (0.7) | **P=<0.0001** | 11 (1.3) | 19 (0.6) | **P=0.0153** |
| **Bipolar disorders** | 17 (2.0) | 24 (0.8) | **P=0.0017** | 13 (1.6) | 35 (1.1) | **P=0.2378** |
| **Unipolar mood disorders** | 149 (17.7) | 263 (8.3) | **P=<0.0001** | 59 (7.2) | 131 (4.2) | **P=0.0003** |
| **Anxiety disorders (including childhood anxiety disorders)** | 57 (6.8) | 104 (3.3) | **P=<0.0001** | 22 (2.7) | 59 (1.9) | P=0.1442 |
| **Personality disorders** | 38 (4.5) | 46 (1.5) | **P=<0.0001** | 25 (3.0) | 50 (1.6) | **P=0.0072** |
| **Alcohol and drug addiction/abuse** | 40 (4.8) | 75 (2.4) | **P=0.0003** | 70 (8.5) | 124 (3.9) | **P=<0.0001** |
| **Attention-deficit/**  **hyperactivity disorders** | 3 (0.4) | 11 (0.4) | **P=0.9474** | 3 (0.4) | 4 (0.1) | **P=0.1503** |
| **Autism spectrum disorders** | 0 (0.0) | 1 (0.03) | **N/A** | 3 (0.4) | 1 (0.03) | **N/A** |
| **Conduct/oppositional defiant disorders** | 1 (0.1) | 1 (0.03) | **N/A** | 0 (0.0) | 2 (0.1) | **P=0.9653** |
| **Learning and**  **coordination disorders** | 4 (0.5) | 2 (0.1) | **P=0.019** | 3 (0.4) | 1 (0.03) | **P=0.0314** |
| **Intellectual disability** | 2 (0.2) | 0 (0.0) | N/A | 0 (0.0) | 1 (0.03) | **N/A** |
| **Other psychiatric disorders** | 81 (9.6) | 225 (7.1) | **P=0.0107** | 36 (4.4) | 112 (3.6) | **P=0.3129** |

P-values in groups, where significance remains similar in the whole data and sub-data are **bolded**
